# A Web-Based 24-H Dietary Recall Could Be a Valid Tool for the Indicative Assessment of Dietary Intake in Older Adults Living in Slovenia

**DOI:** 10.3390/nu11092234

**Published:** 2019-09-16

**Authors:** Matej Gregorič, Katja Zdešar Kotnik, Igor Pigac, Mojca Gabrijelčič Blenkuš

**Affiliations:** 1 Health Survey and Health Promotion Centre, National Institute of Public Health, Trubarjeva 2, 1000 Ljubljana, Slovenia; Matej.Gregoric@nijz.si; 2 Department of Biology, Biotechnical Faculty, University of Ljubljana, Večna pot 111, 1000 Ljubljana, Slovenia; Katja.Zdesar@bf.uni-lj.si; 3 Department of Food Science and Technology, Biotechnical Faculty, University of Ljubljana, Večna pot 111, 1000 Ljubljana, Slovenia; IgorPigac@gmail.com

**Keywords:** dietary assessment, dietary recall, weighed food record, OPEN software, residential homes, older adults

## Abstract

The methodology used in dietary surveys could, to a large extent, follow the instructions of the European Food Safety Authority (EFSA), where 24-h dietary recall (24HDR) is recommended for (sub) population studies. However, it is necessary to examine the suitability of 24HDR for indicative dietary intake in older adults. This study aimed to compare participants’ dietary intakes with the recommendations and to compare dietary intakes derived from a 24HDR using an OPEN web-based application to those obtained from reference weighed food records (WFRs). Forty-nine Slovenian residential home residents completed both assessments, and a comparison with dietary reference values was performed. Estimates from these two methods were compared and the correlations between them were assessed. The findings revealed that dietary intakes derived from the WFR method mostly differed from the recommended intakes. The 24HDR underestimated dietary intake compared to the WFR for 66% of monitored parameters, while 75% of these parameters were correlated, mostly at a moderate level (0.3–0.69). In conclusion, the diets of residential home residents in this study mostly differed from recommendations. Both methods for dietary intake assessment provided comparable results for most of the monitored parameters in expected deviations. A web-based 24HDR could be a valid tool for the indicative assessment of dietary intake in older adults. However, further validations are required.

## 1. Introduction

From a health perspective, older adults are one of the most vulnerable groups. Poor physical health, lack of physical activity, inadequate nutrition, and low skeletal muscle mass are among the most important factors that contribute to the progression of chronic diseases and various acute health problems [1].

Having a proper diet significantly affects the aging process and is the most important element in preventing malnutrition, sarcopenia and frailty [2]. Nutritional requirements in older adults are specific in order to avoid malnutrition, which is considerably higher (30–65%) in hospitalized or institutionalized older individuals [3]. Therefore, monitoring dietary intake and achieving adequate nutrition is fundamental to achieving a proper nutritional status, healthy and active ageing, and successful treatment outcomes in older adults [4].

Nutritional status assessment usually combines anthropometric, biochemical, clinical, and dietary intake data to determine whether an individual or groups of people is/are properly nourished or malnourished. Mini Nutritional Assessment (MNA) is one of the most commonly used screening tools, and is recommended by the European Society for Clinical Nutrition and Metabolism (ESPEN) in geriatric settings to identify vulnerable individuals. The MNA includes standard screening parameters, such as body mass index (BMI) as an indicative measure, weight loss, (reduced) dietary intake and diseases, among others. When BMI is not obtainable in geriatric individuals, calf circumference is recommended as an indicator of body composition within MNA [5]. However, ESPEN guidelines point out that, due to changes in body composition during aging and a reduction in body height, the validity of BMI as a measure of overweightness and obesity is reduced in older people. Therefore, BMI remains a highly controversial measure of obesity and overweightness in that age group [5].

In older individuals who are identified as malnourished with screening, a comprehensive nutritional assessment based on relevant dietary intake methodology should be followed. An important aspect of nutritional assessment is monitoring the individual intake of energy and crucial nutrients, such as proteins, fats, vitamin B6, vitamin B12, folic acid, vitamin D, calcium, antioxidants, selenium and zinc [6]. For these estimates, precise, reliable and detailed quantitative data on food and fluid consumption are needed [7].

Most methods for the collection of food intake information are suitable for older adults in clinical assessment and assessment in population-based studies. However, some methods are not necessarily acceptable for older adults in population-based studies, e.g., online 24-h dietary recall (24HDR) tools, whereby participants with limited internet access may not be able to take part in certain studies. In addition, diminished functionality and cognitive decline may hamper dietary assessment and require a tailored approach to assess dietary intake in older adults [8]. Each of these methods for dietary intake assessment has its own advantages and disadvantages [9]. Consequently, there is no consensus on the method recommended in population studies [10], as well as in clinical settings [11]. A major step forward in this area has been made by the European Food Safety Authority (EFSA), which followed a large-scale validation project [12] and concluded that two non-consecutive 24HDRs are the gold standard for Europe for estimating usual dietary intakes [13]. Furthermore, in recent years, computer-assisted dietary intake assessment tools have been developed to facilitate and simplify recording, assuring a higher level of accuracy and enabling the access of results quickly [14].

Regardless of the used method, self-reported recalls and records underestimate dietary intakes [15]. In a study on hospitalized patients, it was observed that mean energy and protein intakes were significantly underestimated with the 24HDR compared to the reference weighed food record (WFR) [16,17]. Since the accuracy of dietary intake assessment is important in dietary studies, dietary intake methods are often evaluated using one of the more precise methods [15]. Although computer-assisted 24HDR—which is an inexpensive and relatively quick method—provides low response burden and enables multiple recalls, the WFR provides quantified intake data recorded in real time, more detailed information about the intake, and does not rely on a participant’s memory [18,19]. Therefore, the accuracy of the data collected by the 24HDR depends on the accuracy of the reported data and the memory of the respondent. In cases where the foods that are being consumed cannot be quantified by the respondents themselves, different portion size measurement aids should be used to help facilitate quantification [13].

To our knowledge, no studies have yet validated a computer-assisted 24HDR for use in either a national dietary study (including older adults) or a Slovenian clinical/geriatric practice. Therefore, this study aimed to assess dietary intake according to reference values and the suitability of computer-assisted 24HDR for indicative dietary intake in older adults, since the accuracy of dietary intake assessments is an important part of nutritional status assessments.

## 2. Materials and Methods

### 2.1. Study Design and Study Sample

This study was performed as an upgrade of a bilateral research project entitled The Physical Activity and Nutrition for Great Ageing (PANGeA), the methodology of which has been described in detail elsewhere [20]. In brief, the PANGeA project investigated a number of surveyed and measured cross-sectional data related to ageing, including the transposition and implementation of the computer-assisted OPEN based 24HDR in older adults in Slovenia. 

Ethical approval was obtained by the Slovenian Ethics Committee (no. 79/07/13) and was in accordance with the Declaration of Helsinki. All participants were fully informed of the study requirements and were required to provide written consent before participating in the study. 

After initial agreement with the Association of Social Institutions of Slovenia and individual residential homes management, an invitation letter was sent to three representative residential homes in Slovenia in order to invite their residents to participate in the study. A sampling frame included 565 potential respondents, 225 from Residential home 1, 170 from Residential home 2, and 170 from Residential home 3. The inclusion criteria for participants were: (a) Living full-time in a residential home; (b) provided meals at the residential home; (c) older than 65 years; and (d) healthy as defined in the PANGeA criteria [20]. In addition, poor cognitive functions were defined as an exclusion criterion in order to avoid reliance on memory. In total, 110 participants met those criteria, of which 49 agreed to voluntarily cooperate in the study and were therefore recruited during the year 2015. All participants who took part in the study had the opportunity to receive feedback about their results, while medical staff in residential homes had the opportunity to receive key findings from the study.

### 2.2. Data Collection

Residents’ body mass and height were measured using standardized medical equipment and procedures. BMI was calculated by standard principles and assessed using specific BMI cut-off points of <23.0, ≥23.0 and ≥31.0 kg/m^2^ for underweight, normal weight and overweight, respectively [21].

No indication was found in the literature that BMI would significantly affect the results of the study while comparing both employed methodologies, as described below. The main interest in measuring body mass and height in this case was to calculate and assess the energy and nutrients intake by kilogram of body mass.

The dietary intake of each participant was assessed with two non-consecutive 24HDRs, as well as with a 3-day WFR as a reference method. Both were conducted in the same time period. The WFR was chosen as the reference method as its acceptability level of accuracy has previously been validated in several studies related to assessing dietary intake [22,23,24]. A period of three consecutive days in the WFR was performed in order to provide reliable information on usual food consumption and prevent poor compliance if the recording period was too long [23]. In the case of the 24HDRs, two non-consecutive days was sufficient for correction of within-subject variability and for comparison of mean dietary intakes of subgroups [13]. Both dietary assessment methods were performed according to the protocols proposed by the EFSA guidelines for collecting food consumption data [13].

### 2.3. Dietary Assessment

The computer-assisted 24HDR was performed by National Institute of Public Health professionals. The protocol was supported by the Slovenian OPEN web-based application [25,26] and a country- and age-specific picture book for estimating the amount of foods consumed [27]. Trained nutritionists performed face-to-face interviews with each of the participants on two non-consecutive days that were at least one to two weeks apart. In the planning of the interviews, an equal distribution of the different days of the week (including week days and weekend days) was considered. Interviewers collected detailed descriptions of the type and amount of food consumed on the previous day and on food items that could be easily forgotten. The dietary information obtained by the 24HDRs, which followed the structured protocol, was entered directly into the OPEN tool by trained nutritionists. A computer-assisted 24HDR was able to be completed in 20 to 30 min [13]. 

Three-day WFRs were performed and food diaries were completed by trained medical staff, employed in residential homes, only after both 24HDR were conducted with individual participant. Meals were weighted by using professional food scales just prior to being served and after the participants had finished their meals, to assess consumed amounts. WFR is usually time-consuming and labor-intensive for both participant and researcher [22,23].

### 2.4. OPEN Dietary Software

Participants’ energy and nutrient intake data—obtained using both methods—were calculated by National Institute of Public Health professionals using the national OPEN software (Jožef Stefan Institute, Ljubljana, Slovenia), which was upgraded to support 24HDR and dietary records [28]. The calculation procedure and the use of OPEN has been described in detail elsewhere [25]. The mean dietary intakes for energy, water, dietary fibre, 11 macronutrients and 19 micronutrients were determined by OPEN calculations.

### 2.5. Statistical Analysis

The results were statistically analysed using IBM SPSS Statistics 20.0. For all the tests, the level of statistical significance was set to *p* < 0.05.

Distributions of dietary intake data were tested with a Shapiro–Wilk test, when appropriate, to meet the assumption of statistical tests. The dataset of most observed parameters had a normal distribution, so a paired samples *t*-test was used to compare the differences in the intakes between the two 24HDR and WFR [29]. In addition, Pearson’s correlations were computed to test the significance of the relationships between the intake estimates from the two methods [30]. Cut-offs for weak significance between both methods was set at 0.01–0.29 points, for medium at 0.3–0.69 and for strong at 0.7–1.0. The levels of agreement between these two methods were also graphically illustrated for the same parameters using Bland–Altman scatter plots [30,31]. Computational statistical analysis was performed using MedCalc 12.7.1. A Student’s *t*-test was used to determine the differences in mean intakes between genders, while a non-parametric Wilcoxon signed-rank test was used to compare the mean intakes to reference values.

## 3. Results

A total of 49 residential home residents (34 females, 15 males) agreed to voluntarily cooperate in the study and completed both assessments. Table 1 displays the demographic characteristics of the final sample. The mean BMI (±SD) of participants in this study was 30.9 ± 6.7, with no statistical difference between males and females. A total of 4.1% of participants were underweight (BMI < 23.0), while 49.0% were normal weight (BMI ≥ 23.0 and < 31.0) and 46.9% were overweight or obese (BMI ≥ 31.0). 

### 3.1. Comparison of Participant’s Dietary Intakes with the Recommendations

Results from the 3-day WFR revealed that the daily energy and water intake of the subjects was lower than recommended values. The absolute intakes of proteins and dietary fibre were below the recommendations for both genders. The ratio between polyunsaturated fatty acids (PUFA): monounsaturated fatty acids (MUFA): saturated fatty acids (SFA) was 1:2:3 (Table 2).

**Figure 1:**
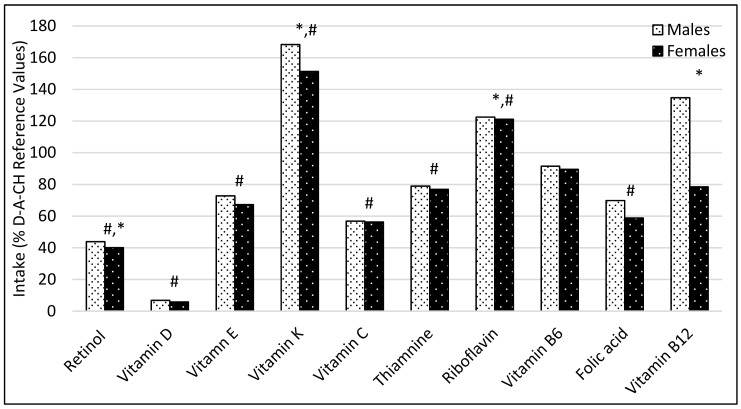
Dietary intake of vitamins expressed as a percentage according to reference values (males (*n =* 15) and females (*n =* 34)). * *p* < 0.05 between males and females. # *p* < 0.05 in comparison with the recommendations.

**Figure 2:**
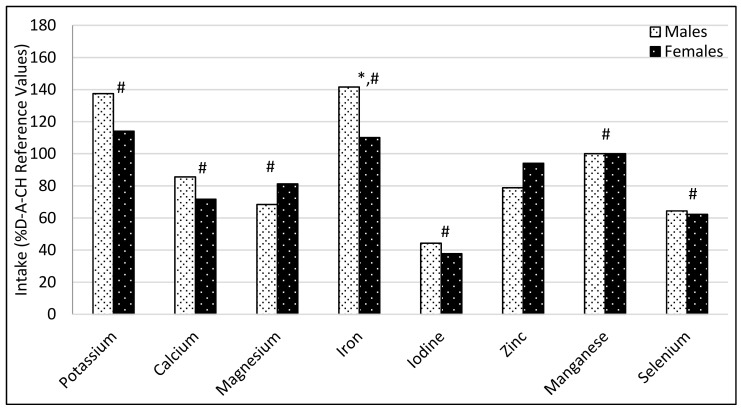
Dietary intake of elements expressed as a percentage according to reference values (males (*n =* 15) and females (*n =* 34)). * *p* < 0.05 between males and females. # *p* < 0.05 in comparison with the recommendations. Results for manganese were inside the reference ranges in terms of both lower or upper values.

As shown in Figure 1 and Figure 2, the mean intake of most vitamins and elements was below the reference values. The lowest intakes were observed in vitamin D, retinol and iodine. The mean intakes of vitamin K, riboflavin, potassium and iron were above the reference values, as well as sodium (2.3 ± 0.7 for male and 2.1 ± 0.6 for female), according to the World Health Organization (WHO) recommendations [34].

### 3.2. Comparison of Dietary Assessment Methods 

The results revealed that the two non-consecutive 24HDRs underestimated dietary intake compared to the WFR for 66% of the monitored nutritional parameters (specifically energy intake, proteins, carbohydrates, dietary fibre, monounsaturated fatty acids, n-3 fatty acids, n-6 fatty acids, vitamin D, vitamin E, thiamine, riboflavin, vitamin B6, vitamin B12, sodium, potassium, calcium, magnesium, iron, zinc, manganese and selenium). However, 75% of these parameters were correlated (specifically energy intake, water, proteins, carbohydrates, total sugars, dietary fibre, total fats, saturated fatty acids, monounsaturated fatty acids, n-3 fatty acids, n-6 fatty acids, cholesterol, retinol, vitamin E, vitamin K, thiamine, riboflavin, folic acid, vitamin B12, calcium, magnesium, iron, zinc and selenium)—mostly in moderate (0.3–0.69) correlation. The ratio between the 24HDRs and the 3-day WFR ranged from 0.67 to 1.48 (Table 3).

**Figure 3:**
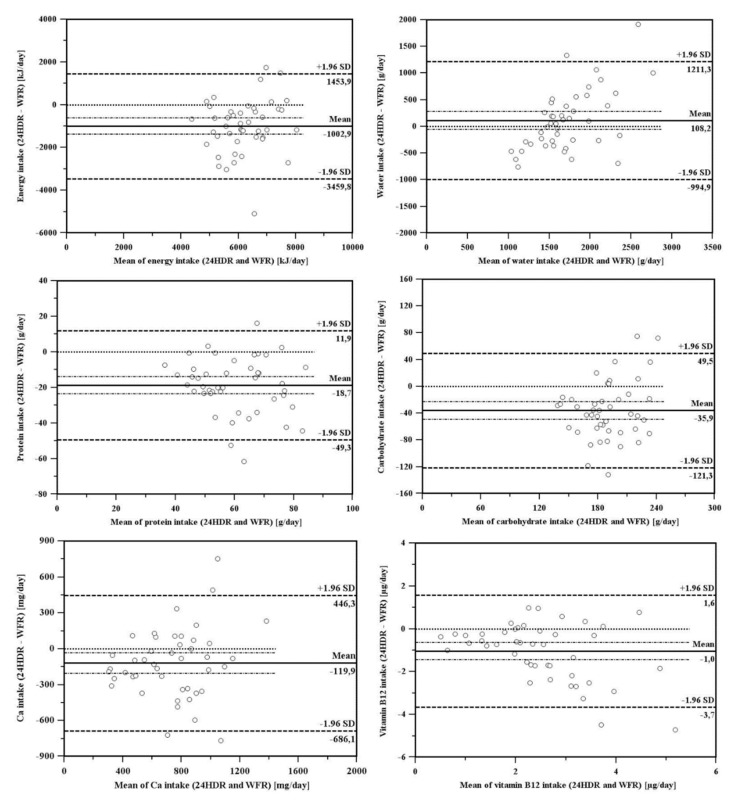
Bland–Altman scatter plots demonstrating the differences between energy, water and nutrient (protein, carbohydrates, vitamin B_12_ and calcium) intake obtained with the 24HDRs and reference WFR methods.

The level of agreement is presented using Bland–Altman scatter plots in Figure 3 for the intake of selected nutrients (i.e., intake of energy, protein, carbohydrates, and water, as well as calcium and vitamin B_12_, which are two of the most important micronutrients for the elderly). The intake of nutrients was obtained from the 24HDRs and the reference method, WFR. The results demonstrated that the majority of the values (more than 92% for all selected nutrients) lay inside the limits of agreement (that is, within 1.96 SD of the mean difference), indicating that a good agreement exists between the two methods. However, the plots for all the selected nutrients (except water) demonstrated that the general bias in mean differences between the reference method (WFR) and the 24HDRs was always negative, suggesting that the 24HDRs slightly underestimated nutrient intake with respect to the reference method. In contrast, the plots for water intake indicated a slightly positive bias in mean differences. In addition, it is indicated that mean differences in water intake assessment with 24HDR and WFR are more pronounced in those participants in which intake of water was much lower or higher than recommended.

## 4. Discussion

To our knowledge, this was the first study to explore the suitability of a two non-consecutive 24HDR method compared to a WFR method among older adults in residential homes. In this study, dietary intake was assessed using methodology and protocols proposed by EFSA guidelines. The dietary intake of residential home residents mostly differed from nutritional recommendations. For this estimation, a web-based 24HDR provided, to some extent, comparable results with the reference WFR.

### 4.1. Comparison of Participant’s Dietary Intakes with the Recommendations

Based on the 3-day WFR, the mean energy intake of participants was lower than the recommended values for a low physical activity level [32]. However, according to the results of other studies, which have found that the physical activity level in residential homes is mostly lower than 1.4, equating to low physical activity in older adults [35,36], this mean energy intake may actually be adequate for the majority of participants.

The proportions of energy derived from proteins, carbohydrates and fats were appropriate and similar to dietary intake patterns observed among older adults in Slovenia [37].

The absolute intake of protein may be inadequate since older adults need higher amounts of protein (1.0–1.2 g/kg body weight) [38,39]. The proportion of energy from fatty acids indicated that the consumption of monounsaturated and polyunsaturated fatty acids was too low, which can be a consequence of unhealthy fats in residents’ diets. Free sugars or equivalent intake was not estimated, as the OPEN database does not support this information. However, the intake of total sugars indicated that this may be a concern.

Most individuals did not meet the requirements for fibre intake, which was also revealed in other studies among older adults, particularly among obese individuals [40]. A low intake of water was also observed; adequate hydration seemed to be a common problem among the older adults due to a weak response to the thirst signal [41].

Among micronutrients, the lowest intakes were observed for vitamin D, retinol and iodine. These findings are in agreement with those from similar studies and can be explained by the fact that most older adults living in institutions are malnourished, frail or overweight, and thus vulnerable to micronutrient deficiencies [42].

### 4.2. Comparison of Dietary Assessment Methods

Looking at the estimates of dietary intakes obtained by both methods, some differences can be found. In general, both methods provided, to some extent, results comparable to other studies [16,17,43,44]. The mean dietary intakes obtained with the web-based 24HDR significantly correlate with those obtained with the WFR method. At the same time, the web-based 24HDR also underestimates the mean dietary intakes compared to the WFR method. Negative 24HDR/WFR ratios were found in other studies, wherein negative ratios were attributed to the omission of snacks and, in particular, lunch and drinks, as well as errors in portion size estimates related to recalled intake [45,46]. Some rare positive 24HDR/WFR ratios can be explained, since participants were frequently prompted in 24HDR to recall missing ingredients, such as water, beverages, coffee drinks, salt and sugar.

This was not a validation study wherein one method is directly compared with another. The aim of the study was to examine two methods by comparing the means of a number of monitored parameters. Furthermore, the WFR method was chosen in this study as the more accurate independent reference method, protected from the risk of under- or overestimation due to recall bias, as seen in other 24HDR studies [15,16].

However, a correlation for most of the monitored nutritional parameters could also be attributed to the web-administrated 24HDR protocol and OPEN application as a highly structured process. In addition, the performance of 24HDR was supported by the picture book, which was specially developed to assist in portion size estimation. Moreover, this approach minimized the differences arising from measurement errors depending on the memory of the subjects on one side and their perception of portion sizes, using picture book on the other [14].

### 4.3. Strengths and Limitations

These two methods were possible to compare with the same software using the same food composition database. The intake record corresponded to the same time period to avoid possible seasonal variations. This study explored the suitability of these methods and dietary tools in older adults in residential homes, thereby adding value since, to date, the majority of those tools were tested and used in adults or younger age groups. According to the results obtained, these were not only suitable for healthy older adults, but also for very old individuals with good cognitive function. This was consistent with the conclusions of some other authors [47], despite the fact that under-reporting in techniques that require interviews is more common among older adults than younger adults. Older individuals with poor health are often not able or willing to participate and need more motivation to participate in such a study [18].

Some limitations of the present study also need to be considered. First, the sample size was small, which limited the detection of significant differences between subgroups. Furthermore, the degree of under- and/or over-reporting of energy intakes in this study was not assessed against the energy need since the extent of physical activity of the participants was not captured. In this context, BMI values also bring out some uncertainties in defining energy needs and nutrition status in older adults. However, a mean BMI around 31 kg/m^2^ indicated a tendency of the participants to be overweight and obese, as is most common in older adults [48,49]. Health risks associated with being overweight are also uncertain at older ages, described as the “obesity paradox” [50]. Consequently, BMI cut-off values for the definition of overweight and obese are still unclear in older adults [51]. Lastly, the food items browser in the web-based 24HDR is open-ended and allows different approaches to identify individual food items, which can reflect slight differences in results, based on differences of the food composition data choice [52].

Nevertheless, some authors pointed out that 24HDR is less sensitive, but sensitive enough for decision-making in clinical practice [16]. For a more accurate assessment of dietary energy and nutrient intake, several days of recalls/records may be required [53] as a further consideration in the described methodological approach. In addition, obtaining high levels of participation in studies of older adults may depend on multiple factors, including the cognitive function of participants [54].

## 5. Conclusions

The diets of residential home residents in this study mostly differed from recommendations. The results should stress the importance of menu planning and carrying out nutritional assessments at the individual level in residential homes. To avoid malnutrition and frailty in institutionalized older adults, nutritional status assessment, including dietary intake assessment, should be a standardized procedure, accessible to each residential home resident.

In addition, both methods for dietary intake assessment provided comparable results for most of the monitored parameters in expected deviations. The 24HDR method, which used OPEN web-based application and age- and country-specific supportive dietary tools, proved to be a valid tool for a relatively quick and simple indicative assessment of dietary intake in older adults. Our results revealed its possible use as an appropriate indicative method for dietary intake assessment in population dietary studies and, to some extent, in clinical practice as a part of a nutritional status assessment.

## Figures and Tables

**Table 1:** Demographic characteristics of participants.

Gender, Age and BMI ^†^	
Male (n)	15
Female (n)	34
Age (Mean, SD *) (years)	75.2 (5.7)
Age range	65–91 years
BMI a (kg/m^2^) (Mean, SD *)	30.9 (6.7)

^†^ BMI—Body mass index; * SD—Standard deviation.

**Table 2:** Comparison of the mean (±SD) dietary intakes from the 3-day WFR and reference values, by gender (males (*n =* 15) and females (*n =* 34)).

	Unit	Male	Female	D-A-CH Reference Values [32]	
				Male	Female
Energy **	kJ	6995 ± 844	6519 ± 1077	8790	7120
Energy	kJ/kg body weight	85 ± 12	86 ± 23	/	/
Water **,^†^	mL	1783 ± 315	1611 ± 337	2250	2250
Proteins **	g	76 ± 14 *	65 ± 15 *	67	57
Proteins **	g/kg body weight	0.9 ± 0.3	0.8 ± 0.3	1.0	1.0
Proteins	% kJ	18 ± 2	17 ± 2	/	/
Carbohydrates	g	210 ± 27	205 ± 35	/	/
Carbohydrates **	% kJ	51 ± 5	54 ± 5	>50	>50
Total Sugars ^‡^	g	65 ± 11	66 ± 23	/	/
Total Sugars ^‡^	% kJ	16 ± 2	17 ± 6	/	/
Dietary Fibre **	g	15 ± 3	16 ± 4	>30	>30
Dietary Fibre **	g/MJ	2.2 ± 0.3	2.5 ± 0.4	>3.1	>3.9
Total Fats	g	57 ± 11	52 ± 13	/	/
Total Fats	% kJ	30 ± 4	29 ± 5	<30	<30
Saturated Fatty Acids	g	21 ± 5	18 ± 5	/	/
Saturated Fatty Acids	% kJ	11 ± 2	10 ± 2	<10	<10
Monounsaturated Fatty Acids	g	14 ± 3	12 ± 3	/	/
Monounsaturated Fatty Acids **	% kJ	7 ± 2	7 ± 1	>13	>13
n-3 Fatty Acids	g	1 ± 0.4	0.8 ± 0.4	/	/
n-3 Fatty Acids	% kJ	0.5 ± 0.2	0.5 ± 0.2	0.5	0.5
n-6 Fatty Acids	g	5.2 ± 1.4	4.8 ± 2.1	/	/
n-6 Fatty Acids	% kJ	2.7 ± 0.7	2.7 ± 1.0	2.5	2.5
Polyunsaturated Fatty Acids	g	7 ± 2	7 ± 2	/	/
Polyunsaturated Fatty Acids **	% kJ	4 ± 1	4 ± 1	>7	>7
Cholesterol **	mg	264 ± 80 *	206 ± 69 *	<300	<300

* *p* < 0.05 between males and females. ** *p* < 0.05 in comparison with the recommendations. ‡ Total sugars are free sugars plus sugars naturally present in foods (e.g., lactose in milk, fructose in fruits) [33]. † Water refers to the total water from beverages and solid foods. Average daily energy requirements calculated as (reference) the basal metabolic rate of individuals with a normal body weight multiplied by the physical activity level value 1.4 [32].

**Table 3:** Differences and correlations in mean (±SD) dietary intakes from the two non-consecutive 24HDR and 3-day WFR.

	Unit	3-Day WFR	2 × 24HDR	Ratio 24HDR/WFR	Paired Samples *t*-Test		Pearson Correlation	
					*t*	*p* *	*r*	*p* **
Energy	kJ	6665.2 ± 1026.9	5739.8 ± 1193.8	0.86	5.367	0.000	0.39	0.005
Water ^†^	ml	1664.1 ± 336.6	1763.8 ± 585.3	1.06	−1.290	0.204	0.42	0.003
Proteins	g	68.9 ± 15.5	51.3 ± 13.2	0.74	8.051	0.000	0.39	0.006
Carbohydrates	g	206.7 ± 32.4	173.8 ± 39.6	0.84	5.526	0.000	0.31	0.033
Total Sugars ^‡^	g	65.9 ± 20.1	59.7 ± 27.6	0.91	1.869	0.068	0.56	0.000
Dietary Fibre	g	15.9 ± 3.8	14.2 ± 5.6	0.89	2.228	0.031	0.40	0.011
Fats (Total)	g	53.6 ± 12.6	50.9 ± 13.6	0.95	1.472	0.148	0.50	0.000
Saturated Fatty Acids	g	19.1 ± 5.4	18.5 ± 6.7	0.97	0.780	0.440	0.33	0.020
Monounsaturated Fatty Acids	g	12.6 ± 3.4	9.9 ± 4.1	0.79	4.528	0.000	0.36	0.012
n-3 Fatty Acids	g	0.9 ± 0.4	0.7 ± 0.4	0.78	3.381	0.002	0.39	0.006
n-6 Fatty Acids	g	4.9 ± 1.9	3.4 ± 1.8	0.69	5.688	0.000	0.47	0.002
Polyunsaturated Fatty Acids	g	6.9 ± 2.1	6.2 ± 3.8	0.90	1.228	0.226	0.09	0.544
Cholesterol	mg	224.4 ± 76.8	203.8 ± 80.6	0.91	1.593	0.118	0.34	0.016
Vitamins								
Retinol	mg	0.4 ± 0.1	0.4 ± 0.3	1.00	−0.713	0.480	0.61	0.000
Vitamin D	µg	1.3 ± 0.7	1.1 ± 0.6	0.85	2.073	0.046	0.36	0.120
Vitamin E	mg	8.2 ± 3.4	6.4 ± 3.2	0.78	3.725	0.001	0.45	0.001
Vitamin K	µg	118.9 ± 57.2	176.4 ± 127.5	1.48	−3.892	0.000	0.60	0.000
Vitamin C	mg	59.4 ± 22.1	61.0 ± 31.9	1.03	-0.332	0.742	0.25	0.090
Thiamine	mg	1.0 ± 0.1	0.8 ± 0.2	0.80	4.380	0.000	0.38	0.008
Riboflavin	mg	1.4 ± 0.4	1.2 ± 0.5	0.86	4.147	0.000	0.58	0.000
Vitamin B6	mg	1.1 ± 0.3	0.9 ± 0.3	0.82	4.114	0.000	0.15	0.338
Folic acid	µg	197.7 ± 54.6	194.4 ± 83.0	0.98	0.341	0.735	0.59	0.000
Vitamin B12	µg	3.0 ± 1.4	2.0 ± 1.0	0.67	5.276	0.000	0.47	0.001
Macro Elements								
Sodium	mg	2134.9 ± 611.4	1745.8 ± 673.2	0.82	3.356	0.002	0.19	0.193
Potassium	mg	2526.8 ± 460.5	2154.5 ± 662.8	0.85	3.779	0.000	0.27	0.060
Calcium	mg	796.3 ± 265.5	686.6 ± 306.1	0.86	2.785	0.008	0.53	0.000
Magnesium	mg	251.1 ± 100.2	212.1 ± 80.4	0.84	2.820	0.007	0.36	0.012
Micro and Trace Elements								
Iron	mg	12.5 ± 3.1	8.8 ± 2.6	0.70	7.313	0.000	0.30	0.036
Iodine	µg	73.9 ± 22.4	81.8 ± 50.2	1.11	−1.112	0.272	0.27	0.060
Zinc	mg	7.3 ± 1.9	5.5 ± 1.9	0.75	6.818	0.000	0.51	0.000
Manganese	mg	4.2 ± 1.6	3.1 ± 1.2	0.74	4.042	0.000	0.07	0.648
Selenium	µg	41.6 ± 12.6	36.6 ± 16	0.88	2.904	0.024	0.50	0.000

*t* Paired *t*-test value. r Pearson’s correlations value. * *p* significant value of *t*. ** *p* significant value of *r*. ^‡^ Total sugars are free sugars plus sugars naturally present in foods (e.g., lactose in milk, fructose in fruits) [32]. ^†^ Water refers to the total water from beverages and solid foods.
